# Injection Molding Simulation of Polycaprolactone-Based Carbon Nanotube Nanocomposites for Biomedical Implant Manufacturing

**DOI:** 10.3390/ma18133192

**Published:** 2025-07-06

**Authors:** Krzysztof Formas, Jarosław Janusz, Anna Kurowska, Aleksandra Benko, Wojciech Piekarczyk, Izabella Rajzer

**Affiliations:** 1 Department of Mechanical Engineering Fundamentals, Faculty of Mechanical Engineering and Computer Science, University of Bielsko-Biala, 43-309 Bielsko-Biala, Poland; 2 Department of Biomaterials and Composites, Faculty of Materials Science and Ceramics, AGH University of Krakow, al. A. Mickiewicza 30, 30-059 Kraków, Poland; 3 Department of Glass Technology and Amorphous Coatings, Faculty of Materials Science and Ceramics, AGH University of Krakow, al. A. Mickiewicza 30, 30-059 Kraków, Poland

**Keywords:** injection molding simulation, polycaprolactone (PCL), carbon nanotubes, 3D printing, filament, biomaterials

## Abstract

This study consisted of the injection molding simulation of polycaprolactone (PCL)-based nanocomposites reinforced with multi-walled carbon nanotubes (MWCNTs) for biomedical implant manufacturing. The simulation was additionally supported by experimental validation. The influence of varying MWCNT concentrations (0.5%, 5%, and 10% by weight) on key injection molding parameters, i.e., melt flow behavior, pressure distribution, temperature profiles, and fiber orientation, was analyzed with SolidWorks Plastics software. The results proved the low CNT content (0.5 wt.%) to be endowed with stable filling times, complete mold cavity filling, and minimal frozen regions. Thus, this formulation produced defect-free modular filament sticks suitable for subsequent 3D printing. In contrast, higher CNT loadings (particularly 10 wt.%) led to longer fill times, incomplete cavity filling, and early solidification due to increased melt viscosity and thermal conductivity. Experimental molding trials with the 0.5 wt.% CNT composites confirmed the simulation findings. Following minor adjustments to processing parameters, high-quality, defect-free sticks were produced. Overall, the PCL/MWCNT composites with 0.5 wt.% nanotube content exhibited optimal injection molding performance and functional properties, supporting their application in modular, patient-specific biomedical 3D printing.

## 1. Introduction

The development of advanced biomaterials and processing techniques is critical to meeting the growing demand for customized high-performance biomedical implants.

A wide range of approaches are used to fabricate 3D biomaterials, which include both conventional techniques [1,2] and advanced ones based on additive manufacturing technologies [3]. Fused deposition modeling (FDM) is one of the 3D printing techniques that provides high flexibility of material selection and processing. In this approach, implants are fabricated by depositing layers of a thermoplastic polymer through a heated nozzle. The polymer filament is loaded into a printer, melted, and then extruded to form a desired shape, layer by layer [4,5,6]. It is worth noting that biomedical-grade polymers are often very costly, and a filament typically comes in spools, which may not be fully utilized when producing small-scale implants, e.g., for bone or cartilage replacement. Furthermore, when one wants to fabricate and optimize new types of materials, devoting time and resources to fabricate continuous filaments in a spool is suboptimal. Hence, the authors propose a novel concept of using smaller polymer sticks that can be connected [7] so as to minimize material waste and streamline the optimization of 3D-printable materials. These sticks can be manufactured via injection molding, and the obtained simulation results define the optimal production parameters of polymers combined with various additives, like carbon nanotubes. In the near future, instead of purchasing entire spools of a specialized filament, it may be possible to assemble sets of modified sticks tailored to specific patient requirements. Compared to traditional FDM approaches, the proposed method introduces a modular filament stick system tailored for biomedical applications. FDM (fused deposition modeling) remains one of the most widely used rapid prototyping methods due to its reliability and simplicity. However, most medical-grade materials are either costly or require complex granulate processing, which limits flexibility and accessibility. Moreover, commercial FDM printers often rely on proprietary filament spools, which are suboptimal when only small volumes are needed for patient-specific implants. To overcome these limitations, our approach leverages injection-molded PCL/CNT filament sticks that can be joined and used in standard FDM printers. Such a solution minimizes material waste and facilitates testing different composite formulations without the need for entire spool production. Importantly, this modular approach also opens new opportunities for scaffold design in osteochondral defect repair, as it allows for the quick substitution and stacking of different material segments within a single printed structure. In contrast to conventional methods that often yield scaffolds with random internal architecture, our method supports higher repeatability and design control, which are essential for analyzing the influence of microarchitecture on cellular responses.

Currently, simulations of injection molding processes are extensively applied in industry [8,9]. In the preliminary design phase, simulations are instrumental in identifying potential defects in mold geometry and flow behavior, thus significantly reducing the likelihood of costly tool modifications and production delays [10]. Commercially available CAE platforms (e.g., Autodesk Moldflow, Moldex3D, and Solidworks Plastics) facilitate detailed analyses of key processing parameters, such as melt flow, cooling rates, and pressure distribution. Thereby, they replace empirical trial-and-error procedures with data-driven optimization strategies [11]. Still, there is a noticeable lack of research focused specifically on simulating the injection molding of biomedical components. Additionally, simulation platforms often lack comprehensive material data regarding biomedical polymers for medical applications.

In our previous work, we developed a material model for polycaprolactone (PCL), based on the data obtained from literature, and implemented it into the Solidworks Plastics simulation environment to analyze the injection molding process [7].

Poly(ε-caprolactone) (PCL) is a synthetic semicrystalline aliphatic polyester that has been extensively investigated in biomedical research due to its exceptional biocompatibility, biodegradability, and favorable physicochemical properties [12]. PCL has a relatively low melting point of about 60 °C, which makes it easily processable via methods like extrusion, electrospinning, and 3D printing [13,14]. This thermal behavior significantly enhances its applicability in biomedical device manufacturing, spanning from controlled drug delivery systems, through orthopedic implants and bone regeneration scaffolds, and ending at substrates for engineering various tissue types [15,16]. Its slow degradation rate under physiological conditions, combined with minimal inflammatory response, renders it particularly suitable for long-term implantable devices. Moreover, its mechanical properties can be finely tuned through copolymerization or blending with other materials, allowing for the development of application-specific solutions [12].

The objective of our former work was to assess how processing conditions and mold geometry affected the efficiency of producing PCL filament sticks intended for the subsequent 3D printing of biomedical implants. Thermal and rheological properties gathered from published sources were incorporated into the software’s material database to enable accurate simulation of the molding behavior. The study investigated the effects of key injection molding parameters (i.e., melt temperature, injection time, packing time, and packing pressure) on the final geometry of the molded polymer sticks.

In this extended study, we introduced multi-walled carbon nanotubes (MWCNTs) at three concentrations of 0.5%, 5%, and 10% into the PCL matrix to examine the influence of nanoparticle reinforcement on the injection molding behavior and quality of the resulting nanocomposite filament. Polymers reinforced with carbon nanotubes (CNTs) are increasingly attracting attention in various fields of research and industry due to their superior mechanical strength, good electrical conductivity, and functional properties that meet the stringent requirements [17,18,19]. Consequently, they are also becoming more and more prominent in 3D printing. One example of commercially available solutions for special applications is PETG (polyethylene terephthalate glycol) polymer modified en masse with CNTs. Such electrostatic discharge-safe (ESD) filaments can be used to fabricate casings of electronic components. This solution is offered by the Fiberon company [20]. Other examples are various products offered by Mechnano, wherein oxidized CNTs are bonded with a dispersing agent (polyethylene oxide or polyvinyl alcohol) to improve their dispersion and then added to a polymer matrix for the successive 3D printing of ESD materials [21]. Herein, the preferred concentration of CNTs is claimed to be between 0.01% to 5% by weight.

While there are some commercial CNT-modified 3D-printable materials for various industrial applications, reports concerning cytocompatible biomedical materials are restricted to scientific literature. As mentioned earlier, this is mostly because of the costs associated with optimizing compositions from medical-grade materials. Meanwhile, fabricating available 3D-printable cytocompatible materials modified en masse with CNTs could distinctly benefit the broad biomedical field. CNTs can improve electrical and thermal conductivities, grant materials antibacterial and anticancer properties, and positively affect cellular response [22,23,24,25,26]. At the same time, 3D printability creates almost limitless options for recreating challenging tissue architectures or miniaturizing healthcare electronic devices.

Three aspects are essential when optimizing CNT-modified nanocomposites for 3D printing. (1) The CNT concentration has to overcome the percolation threshold. (2) The CNT dispersion, which directly affects the outcome mechanical and electrical properties, must be sufficient, as the better the dispersion, the lower the concentrations necessary. (3) The CNT impact on materials’ processability must be accounted for. Namely, with too high of a concentration, materials become brittle and/or unable to solidify; they also have unprintable rheological properties [27,28,29]. For the well-dispersed CNTs, a concentration below 5% (and even below 1%) already grants materials with sufficient electrical conductivity while not compromising mechanical properties. For example, Kim et al. found that concentrations below 4% created electrically conductive cytocompatible elastomers that could be utilized to fabricate elastic electronics and mechanosensors [30]. Higher concentrations resulted in significant cytotoxicities. Fortunati et al. revealed the enhanced electrical and thermal properties and good cytocompatibility of 1% CNTs added with 15% of silver into PCL. Dottori et al. discovered the significantly enhanced electrical conductivity of PCL at CNT concentrations ≤ 1% [31].

From the economic point of view, lower CNT concentrations that still provide required features are definitely more advantageous. In this comprehensive investigation, we analyzed the impact of three multi-walled carbon nanotube (MWCNTs) concentrations in the PCL matrix on nanoscale reinforcement via injection molding and the resulting quality of the extruded nanocomposite filaments. The assumed go-to concentration of CNTs was 0.5%, but higher concentrations of 5% and 10% were also analyzed for the comparison’s sake. To enable accurate simulation of the molding behavior, relevant thermal and rheological properties of CNTs were integrated into the software database, based on literature.

The primary objective was to evaluate the impact of varying CNT contents on the injection molding performance of PCL/CNT nanocomposites. Particular attention was given to key injection molding parameters, such as injection time, packing pressure, packing duration, flow front temperature, frozen regions extent at the cycle end, and final part temperature. We also investigated the parameters’ influence on dimensional fidelity and the structural integrity of the molded components, thus assessing their processability and potential biomedical applicability.

## 2. Materials and Methods

The injection molding simulations and the material model development were carried out using SolidWorks Plastics 2021 software (version 29.3.0.0059, Dassault Systèmes, Paris, France). This specialized CAE platform designed to simulate and optimize polymer injection accurately predicts flow dynamics, cooling behavior, and potential defects, facilitating the development of reliable processing conditions for nanocomposite materials. The material model, developed in our previous study, was based on the PCL polymer CAPA^®^ 6500 (Ingevity, SC, USA), with a molecular weight of 50 kDa. All necessary thermal and rheological data for the simulations were sourced from literature and imported into the SolidWorks Plastics material database [7]. For our simulations and successive experimental trials, we chose the NC3100 multi-walled carbon nanotubes, supplied by Nanocyl (Sambreville, Belgium). Our literature survey revealed that the NC3100 [24,32] and NC7000 [33,34] products are often employed in literature to produce 3D-printed materials. NC3100 is a research-grade product of a very high purity (carbon atom content > 95%), while NC7000 is an industrial-grade product of a slightly lower purity (approx. 90%). Since our target application is biomedical, we selected materials with lower amounts of impurities—NC3100. Prior to this study, we also had hands on experience with the NC3100 product we used to fabricate 3D-printable and electrically conductive polydimethylsiloxane-based materials for the in vitro electrical stimulation system [35]. Table 1 presents the data related to multi-wall carbon nanotubes. The complete filament mold design, based on the part model, was created using Siemens NX CAD software. A simplified CAD model was generated from the full CAD assembly of the mold.

To validate the simulation process, PCL stick filaments were produced using a Babyplast 6/10P (Rambaldi, Molteno, Italy) injection molding machine. To be suitable for 3D printing, a filament in stick form must meet several key criteria. It should have a uniform diameter along its entire length and be free from external imperfections, such as surface bubbles or voids. Sticks must be able to fuse seamlessly during a printing process without additional chemical agents. Large agglomerates can clog the nozzle, while internal voids may cause fluctuations in the filament diameter while feeding it into the printer, potentially preventing it from passing through the extruder mechanism.

The mold was manufactured from chrome-molybdenum 1.2316 X38CrMo16 cold work tool steel. The mold dimensions were 75.0 mm × 75.0 mm × 76.0 mm (height), while the molded parts measured 60.6 mm (length along the sticks) × 57.8 mm (width across the sticks) × 23.0 mm (height). The process parameters (mold temperature, melt temperature, and injection pressure) are summarized in Table 2. Polycaprolactone pellets (80 kDa) were purchased from Sigma-Aldrich, Gillingham, UK. NC3100™ multi-wall carbon nanotubes used in the study were purchased from NANOCYL^®^. These were unfunctionalized CNTs, with >95% of carbon atoms and <5% metal oxide impurities (from the metal catalyzer used in chemical vapor deposition (CVD) employed for their production). Their average diameter was 9.5 nm, while their average length was 1500 nm. The rest of the parameters can be found in Table 1. Amongst different CNT suppliers, Nanocyl offers products of highly repetitive qualities, since their products are also available at different resellers, including Merck. (NC3100™ is the same product as 755,133 from Merck.) This is reflected in nearly 9000 scientific articles citing the usage of Nanocyl carbon nanotubes. As a comparison, another popular vendor, NanoAmor, was cited by 2500 articles (source: Google Scholar).

The nanocomposite was prepared by mixing poly(ε-caprolactone) (PCL) with 0.5 wt% carbon nanotubes (CNTs) mechanically. First, carbon nanotubes were dispersed into the PCL powder using a mechanical mixer to achieve a preliminary distribution. The mixture was then homogenized and rolled at 190 °C. The material was rolled with a force that facilitated the uniform dispersion of the nanotubes within the polymer matrix. The rolling process lasted approx. 10 min, ensuring adequate melting and mixing without thermal degradation. After homogenization, the blend was cooled, cut into small pieces, and processed into granules suitable for injection molding. To verify the nanocomposite uniformity, macroscopic examination of the molded parts was performed. No visible phase separation was observed, indicating that the nanotubes were well dispersed within the PCL matrix, yet small CNT agglomerates were present. Additionally, the successful filaments fusion in FDM printing trials further confirmed the material homogeneity. In summary, mechanical mixing combined with thermal homogenizing via rolling ensured a uniform distribution of CNTs in the polymer matrix, suitable for molding and printing processes.

For mechanical characterization, specimens with a dog-bone geometry were fabricated via injection molding. The processing parameters were similar to those used in the stick manufacturing process. To characterize the mechanical behavior, both ultrasonic testing and tensile measurements were conducted. Ultrasonic testing was carried out using a CT-3 material tester (Unipan-Ultrasonics, Warsaw, Poland), focusing on wave propagation in the transverse direction (across the specimen’s width). Measurements along the longitudinal axis were found to be less reliable due to reduced stiffness and the influence of the applied force. The measurements were carried out using a through-transmission technique with a pair of 1 MHz transducers (transmitting and receiving heads). An adhesive tape was used as a coupling medium. For each sample, at least four independent measurements of the longitudinal wave transit time were recorded, from which the average velocity and standard deviations were calculated. During the tests, a pulse amplitude of 600 V was applied with a gain of +20 dB and a repetition frequency of 1 Hz.

Mechanical properties were evaluated using a universal testing machine (Hegewald & Peschke, Nossen, Germany) in accordance with ISO 527-1:2019 [41]. During the determination of Young’s modulus, within the strain range of 0.05% to 0.25%, the test speed was set to 1 mm/min. For the remaining part of the test, the crosshead speed was set to 10 mm/min. All the tests were conducted under ambient conditions (~20 °C). For each material variant, six specimens were tested, each with an approximate thickness of 2 mm and a width of 2.04 mm. The stress–strain curve was used to determine Young’s modulus via the secant method within the specified strain range. The results for Young’s modulus, tensile strength, and elongation at break were reported as mean values along with their respective standard deviations.

## 3. Results

**Figure 1:**
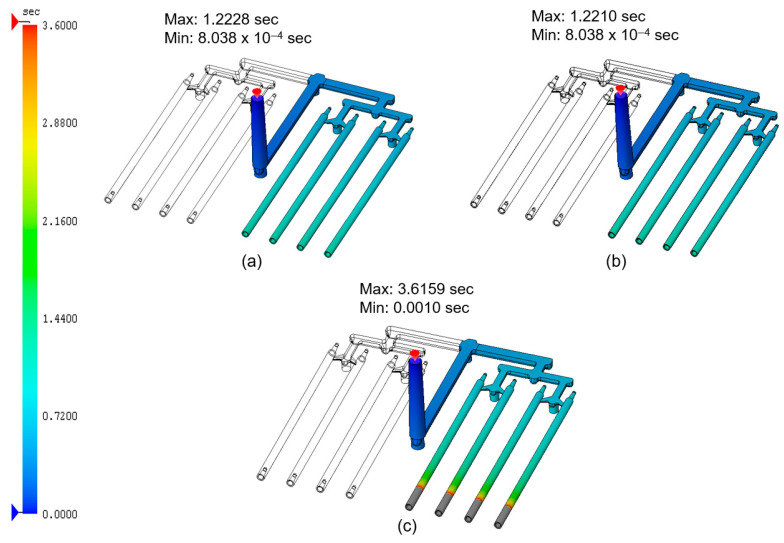
Total filling time for PCL samples containing 0.5, 5, and 10 wt.% carbon nanotubes: (**a**) PCL_0.5; (**b**) PCL_5; (**c**) PCL_10.

Figure 1 illustrates the total fill time for the PCL material containing 0.5%, 5%, and 10% carbon nanotubes, respectively.

As shown, the total fill time for the analyses of the 0.5 and 5% CNT contents (Figure 1a and Figure 1b, respectively) was approximately 1.2 s. In contrast, an analysis for 10% CNTs (Figure 1c) resulted in a significantly longer fill time of 3.6 s, and the sticks volume was not completely filled. It is noteworthy that the simulation was performed for the runner system and only on one side of the stick assembly. In the real world, the mold consists of two parallel sides, as indicated by the transparent outlines in the figure.

**Figure 2:**
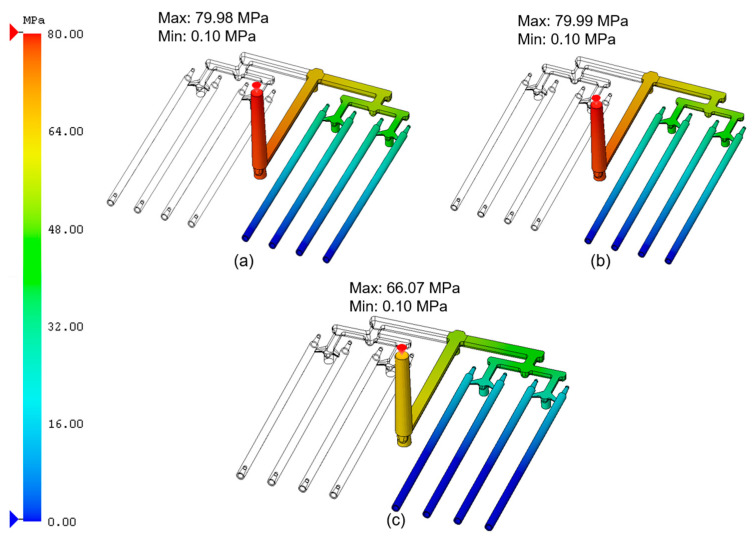
Pressure at the packing switch time for PCL samples modified with 0.5, 5, and 10 wt.% CNTs: (**a**) PCL_0.5; (**b**) PCL_5; (**c**) PCL_10.

Figure 2 shows the pressure value at the moment of switching between the first injection pressure (fill) and the second injection pressure (pack). In the analyses for PCL_0.5 (Figure 2a) and PCL_5 (Figure 2b), the values reached a maximum of approx. 80 MPa. For the analysis of PCL_10 (Figure 2c), it was around 66 MPa. It was evident that pressure was the highest at the beginning of the filling process (runner system), and it decreased as the mold cavity was being filled. In the case of the PCL_10 sample (Figure 2c), a low-pressure zone covered almost the entire volume of the stick set, which was an undesirable effect. The actual filling pressure selected for the process should equal or exceed the indicated maximum value (80 MPa), while the packing pressure may be lower. During the actual injection molding process, it is crucial to account for the pressure intensification ratio (PIR). This parameter describes a relationship between the injection pressure and the hydraulic pressure, i.e., the oil pressure acting on the piston of the injection molding machine. While simulation analyses may suggest relatively high injection pressures, due to this ratio, the actual pressure recorded on the machine can be significantly lower. Typically, PIR values range between 7 and 15. For this reason, appropriate process pressure settings should be selected experimentally, based on the visual inspection and the molded parts quality [7].

**Figure 3:**
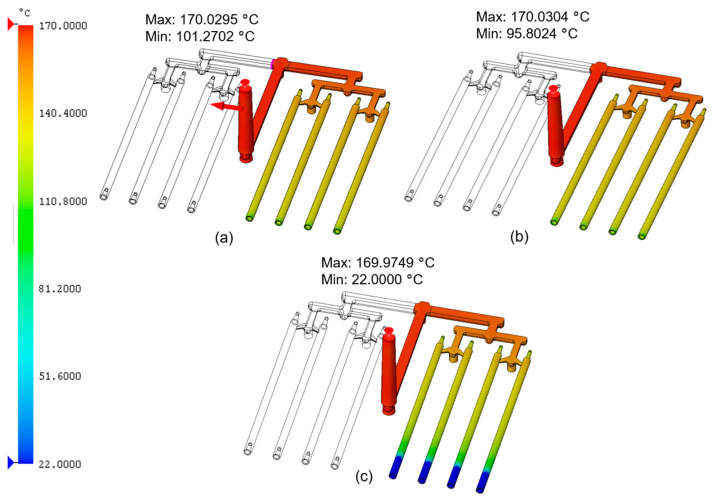
Flow front temperature during the injection process for PCL samples modified with 0.5, 5, and 10 wt.% nanotubes: (**a**) PCL_0.5; (**b**) PCL_5; (**c**) PCL_10.

Figure 3 presents the flow front temperature distribution during the filling stage for the analyzed PCL samples. The maximum melt front temperature in each case was approx. 170 °C. As the cavity filled and the material cooled, the temperature gradually decreased. For (a) (PCL_0.5), the temperature dropped to around 101 °C, and for (b) (PCL_5), it reached approx. 96 °C. In the analysis of (c) (PCL_10), the temperature dropped to ambient conditions. This indicated that PCL_10 reached the minimum melt temperature (58 °C) earlier than expected, prematurely terminating the filling process. Such a phenomenon may have resulted from two facts: the improved thermal conductivity of the sample with the highest share of CNTs and the flow’s physical obstruction posed by the CNT network.

**Figure 4:**
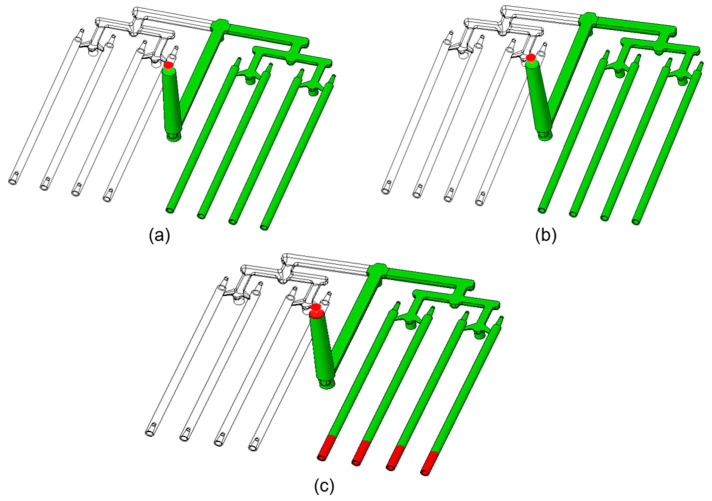
Frozen areas at the injection process end for PCL samples modified with various wt% nanotubes: (**a**) PCL_0.5; (**b**) PCL_5; (**c**) PCL_10.

Figure 4 shows the frozen areas at the end of the injection process for the tested PCL samples with different nanotube contents. These results complement the previous analysis of the flow front temperature. The red color marks the locations of frozen areas, defined as the regions where the temperature fell below 58 °C. No frozen areas were observed in the analyses of PCL_0.5 (Figure 4a) and PCL_5 (Figure 4b), indicating the proper material flow and cavity filling. In the PCL_10 analysis, the stick tips were frozen.

**Figure 5:**
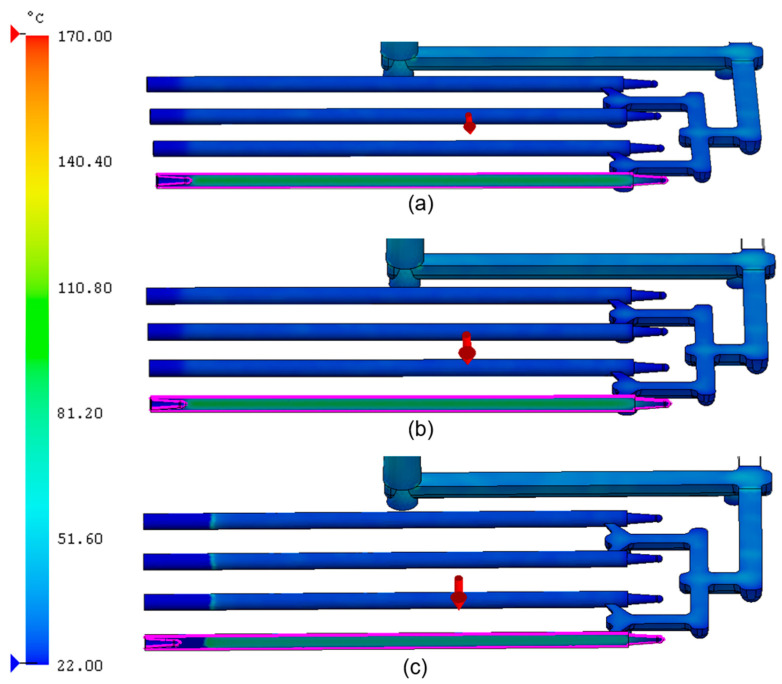
Temperature distribution at the injection process end (clipping section through sticks): (**a**) PCL_0.5; (**b**) PCL_5; (**c**) PCL_10.

By the end of the process (following the initial cooling phase), the outer regions of the molded sticks (the shell) reached the solidification temperature, but the core remained at an elevated temperature (Figure 5). Such results were similar across all the sample types. Therefore, additional post-molding cooling in open air was necessary to provide uniform temperature distribution across the entire cross-section and to bring the part to an ambient temperature.

**Figure 6:**
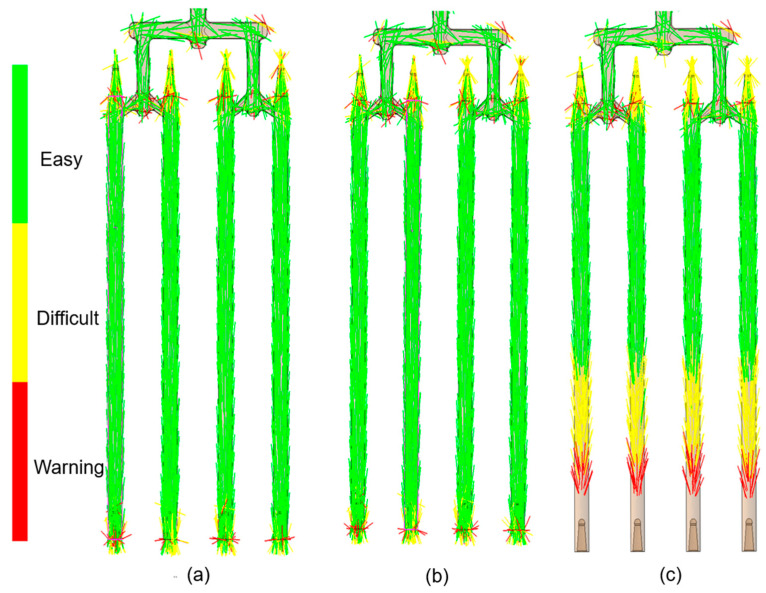
Simulated fiber orientation in composite sticks with CNT contents of (**a**) 0.5 wt.%; (**b**) 5 wt.%; and (**c**) 10 wt.%.

**Figure 7:**
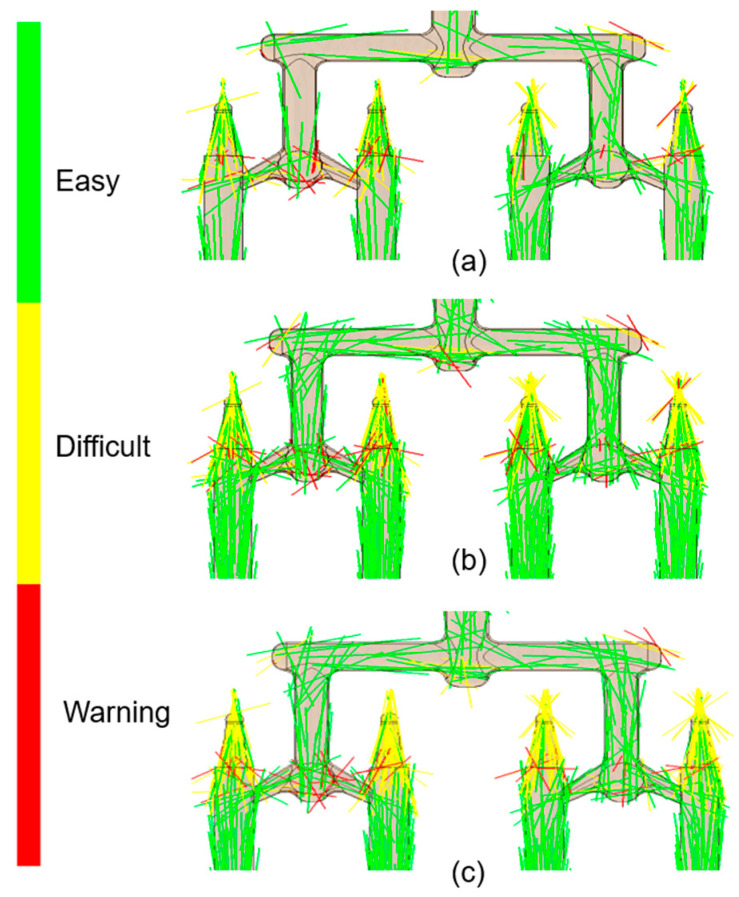
Top view of the fiber orientation in molded sticks: (**a**) PCL_0.5; (**b**) PCL_5; (**c**) PCL_10.

**Figure 8:**
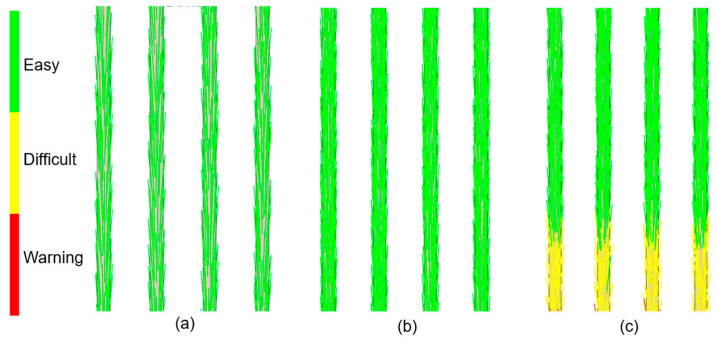
Fiber orientation—sticks’ middle cross-sectional view: (**a**) PCL_0.5; (**b**) PCL_5; (**c**) PCL_10.

**Figure 9:**
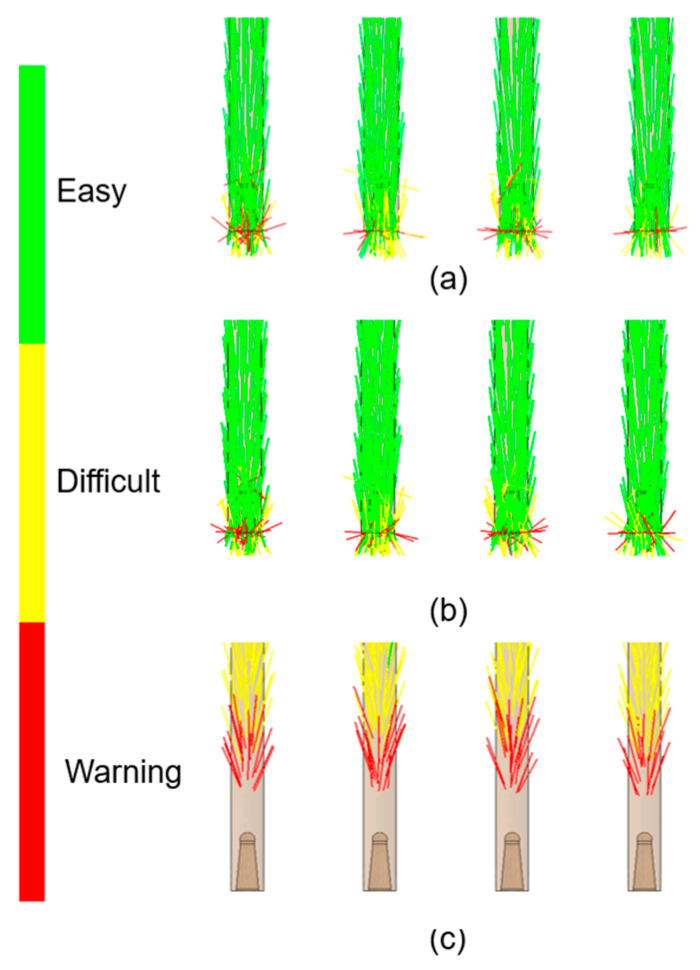
Fiber orientation—sticks’ bottom view: (**a**) PCL_0.5; (**b**) PCL_5; (**c**) PCL_10.

The nanotubes orientation corresponded closely to the flow patterns within the mold cavity (Figure 6, Figure 7, Figure 8 and Figure 9). In the regions with a larger cross-sectional area, the laminar flow led to fiber alignment parallel to the flow direction. Conversely, in narrow channels, fibers tended to orient transversely or randomly to the flow. This relationship was particularly evident in constricted transitions between the runner system and the molded sticks, as well as at their distal ends. In the analysis (Figure 6c and Figure 9c), the flow stopped, which corresponded to the terminated fiber distribution within that region.

To facilitate comparison and improve readability, all the key results are summarized in Table 3.

**Figure 10:**
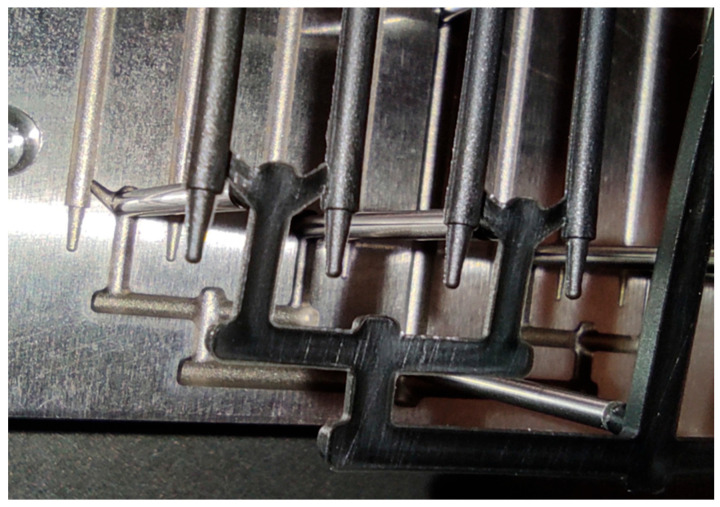
Injection-molded part containing 0.5 wt.% CNTs. Flash and overpacking clearly visible.

The experimental injection molding tests were conducted using the parameters assumed in the simulation for the PCL sample containing 0.5 wt.% CNTs. The resulting molded part defects (e.g., flash formation and overpacking) indicated that the initial processing parameters were still to be optimized (Figure 10).

To address this issue, the injection parameters were slightly modified. Namely, the injection pressure was reduced, and the melt temperature increased (Table 4).

**Figure 11:**
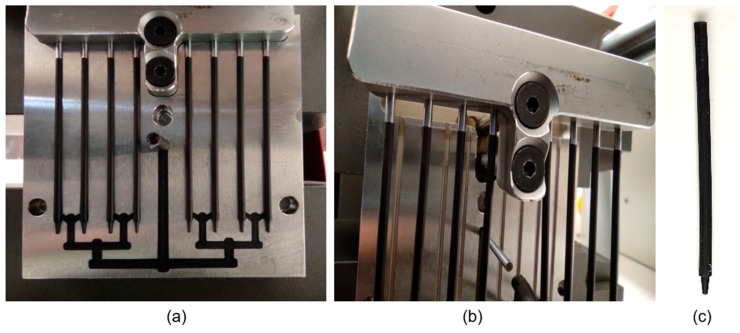
Molded part after parameter adjustments: (**a**) part inside the mold after modified settings injection; (**b**) mold’s upper section with fully formed sticks, complete filling, and no premature solidification; (**c**) final filament—a stick containing 0.5 wt.% CNTs.

These changes improved the material’s flow behavior and made mold cavities fill completely. Due to modifications, fully formed sticks were obtained (Figure 11), with no signs of premature solidification or material freezing. The optimized parameters led to the high-fidelity replication of the intended geometry, confirming the validity of the simulation trends and the importance of experimental feedback.

**Figure 12:**
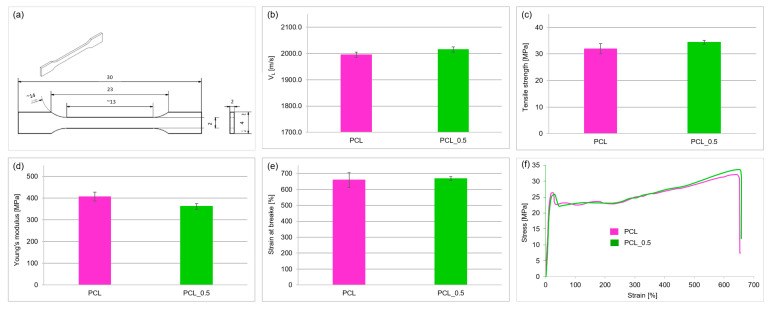
Mechanical test results: (**a**) geometry of the dog-bone specimens utilized in the experiments; (**b**) longitudinal ultrasonic wave velocity; (**c**) tensile strength; (**d**) Young’s modulus; (**e**) strain at break; (**f**) representative stress–strain graphs.

Figure 12 presents the mechanical testing results of the fabricated composite materials. The addition of 0.5 wt% CNTs to polycaprolactone in the fabricated materials slightly increased the average longitudinal ultrasonic wave velocity (1995 m/s for pure PCL and 2015 m/s for PCL_0.5). However, the 1% difference can be considered within the margin of measurement error.

The addition of 0.5 wt% nanotubes to PCL caused an 11% decrease in Young’s modulus, from 407 MPa to 362 MPa (Figure 12d). The addition of nanotubes minimally increased the tensile strength, strain at failure, and average ultrasonic wave velocity; however, these values remained within the margin of measurement error.

**Figure 13:**
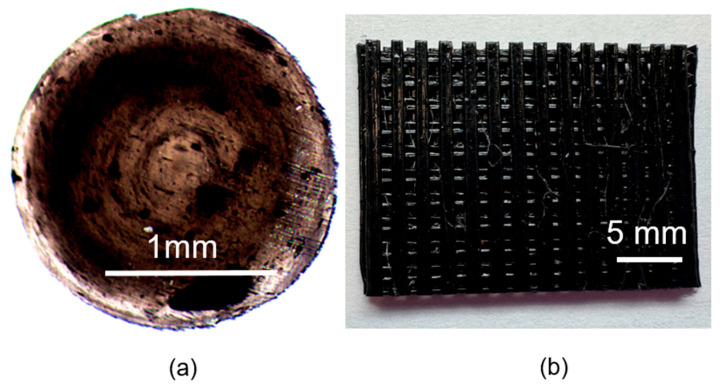
(**a**) Cross-section of the filament observed under a microscope—visible nanotube agglomerates. (**b**) Printed scaffold fabricated using a commercially available 3D printer.

After producing the filaments in the form of sticks using injection molding, they were subjected to microscopic observations (Figure 13), including a cross-sectional analysis. Internal defects may form during the molding of filament sticks and may not be visible through external inspection. These defects could include voids, air bubbles, or nanotube agglomeration resulting from the compounding process. Nevertheless, preliminary FDM printing trials demonstrated that the fabricated filaments could be successfully fused and used to print scaffolds (Figure 13b). This was supported by macroscopic images of the scaffold and stick cross-sections (Figure 13a), which indicated that any agglomerates present did not interfere with the printing process or the formation of the scaffolds.

## 4. Discussion

This study examined how varying concentrations of multi-walled carbon nanotubes (MWCNTs) influenced the injection molding behavior of polycaprolactone (PCL). In our simulations, we modeled the usage of NC3100 multi-walled carbon nanotubes. These CNTs were previously successfully employed in our studies, as well as in works by other authors. We experimentally found their percolation threshold to be at 0.25% in PDMS-based, 3D-printable materials. Postiglione et al. [32] found the percolation threshold in PLA-based materials at the 0.67% CNT concentration of the same CNTs. Goncalez et al. [24] found that the 0.1% concentration of NC3100 was sufficient to grant the acrylic photocurable polymers electrical conductivity and boost their mechanical properties. Both features were further enhanced by increasing the CNT concentration up to 1.5%. Wan et al. used NC7000 to fabricate electro-responsive and 3D-printable composites of D,L-lactide-co-trimethylene carbonate, obtaining electrically conductive and mechanically enhanced materials at 5 and 10% concentrations. The 10% concentration was then used in extrusion-based photocurable 3D printing. Other examples of 3D-printed materials modified en masse with carbon nanotubes involved using different CNTs. In one example, Plasticyl ABS1501, i.e., a commercial pellet of acrylonitrile butadiene styrene (ABS) modified en masse with NC7000, was employed for fabricating CNT-modified filaments [42]. Results found a nine-order-of-magnitude reduction in the sample’s resistivity and a significant increase in mechanical properties. Both values were directly correlated with the CNT content. Spinelli et al. [43] used CNTs of dimensions similar to NC3100 (from Times Nano, China) to fabricate 3D-printable and electrically conductive PLA-based filaments for FDM-based 3D printing. The reported percolation threshold was at 1.5% of CNTs. (Lower concentrations were not tested.) Tsang et al. [44] used CNTs of dimensions similar to NC3100 (from Conjutek, Taiwan) to fabricate 3D-printable extrudable inks based on polyvinyl alcohol (PVA). The 3D printing was followed by the solvent exchange—the water in the hydrogel was replaced with a deep eutectic solvent to obtain stable materials. CNT concentrations of 7, 8, and 9% in the hydrogel were used. All the tested materials were electrically conductive and had good mechanical properties.

This research focused particularly on the nanocomposites’ suitability for fabricating modular filament sticks for biomedical applications. The simulations revealed that the increasing CNT content significantly altered the processing behavior of the PCL matrix. The 0.5% and 5% CNT samples exhibited similar total filling times (approx. 1.2 s), with relatively stable temperature and pressure distributions, which suggests their favorable processability. In contrast, the 10% CNT sample demonstrated a tripled fill time and incomplete cavity filling. This effect was attributed to enhanced thermal conductivity (caused by a higher CNT content), which promoted rapid cooling, premature solidification, and limited flow extension. Additionally, the potential CNT agglomeration and increased melt viscosity impaired the flow and disrupted fiber orientation patterns (Figure 6c and Figure 9c), particularly at the narrow tips of the molded sticks. The samples with 0.5% and 5% CNTs reached peak pressures of approx. 80 MPa, whereas for the 10% CNT, it was only about 66 MPa. In the 10% CNT sample, the pressure drop across the cavity was more pronounced, resulting in extensive low-pressure regions that hindered proper mold filling. Moreover, the flow front temperature analysis indicated that the 10% CNT sample solidified prematurely, which resulted in extensive frozen regions at the distal ends of the mold cavity. The fiber orientation analysis showed that the laminar flow aligned CNTs with the melt direction in wide regions, while the constricted flow disrupted this alignment—again, especially in the 10% CNT material.

The experimental injection trials were conducted using the simulation-derived parameters for the 0.5% CNT. The initial molded parts exhibited flash and short shots (Figure 10), suggesting slight deviations between the modeled and real-world behaviors. The differences likely stemmed from machine-specific factors, such as pressure intensification and shear effects unaccounted for in the simulation. After modest parameter adjustments (i.e., injection pressure reduction and melt temperature rise), the fully formed sticks were obtained (Figure 11a–c), fully validating the simulation predictions and processability of the 0.5% CNT formulation.

A comparison of key processing parameters showed a high level of agreement between the predicted and experimental results, particularly in terms of cavity filling and the absence of premature solidification. Nonetheless, minor deviations were observed, especially in the recorded pressure values and part surface quality. These were attributed to the factors beyond the simulation’s scope, such as pressure intensification ratio (PIR), shear gradients specific to the injection system, and the variable material flow under real processing conditions. Another important limitation of the simulation model lay in the material data. The thermal and rheological properties were derived from literature sources for CAPA^®^ polycaprolactone (Mw = 50 kDa), rather than directly measured for the experimental-grade PCL (Sigma Aldrich, Mw = 80 kDa) and its nanocomposite blends. The use of generic or approximated input parameters may have affected the quantitative accuracy of the model. Therefore, future works should incorporate experimentally determined properties of both the base polymer and CNT-reinforced formulations to improve model fidelity.

This limitation is in line with the challenges reported in previous studies [45], where high simulation accuracy was closely linked to the precisely matched material parameters. Thus, refining the simulation model with directly measured, formulation-specific data is strongly recommended as a next step.

The performed mechanical tests indicated that the introduction of carbon nanotubes did not adversely affect the mechanical properties of the resulting composite materials. The addition of 0.5% nanotubes did not disrupt the filament formation process via injection molding, and the mechanical parameters of the produced sticks were suitable for use in 3D printing. Minor agglomerates formed during the injection molding process in the filaments did not negatively impact the scaffold printing process.

The study results indicated that the 0.5 wt.% CNTs provided an optimal balance between improved functionality and stable processing. This is consistent with literature reports, which frequently identify low CNT loadings (<1%) as ideal in terms of electrical conductivity, thermal enhancement, and bioactivity without compromising cytocompatibility or inducing processing complications [27,28,29,30,31]. Fortunati et al. reported enhanced electrical and thermal performances of PCL composites at 1% CNTs, while Dottori et al. observed significant conductivity improvements at concentrations as low as 0.5% [26,31]. Kim et al. emphasized that higher concentrations may induce cytotoxic responses, further supporting the preference for minimal effective CNT dosages in biomedical contexts [35]. From an economic perspective, low CNT concentrations reduce both material costs and the risk of nozzle clogging or material degradation during extrusion-based 3D printing. Such factors are relevant with regard to the proposed modular filament concept, where short sticks (produced via injection molding) can be assembled for personalized implant fabrication without committing to entire spools. With the use of 0.5% CNTs, the material remains compatible with standard 3D printing hardware. At the same time, it offers crucial functional advantages (e.g., antimicrobial potential, improved conductivity) and processability, ensuring consistent mold filling and structural integrity.

This study introduced a practical and accessible methodology for producing 3D-printable biomedical filaments without reliance on specialized granule-based printers. While standard FDM setups are often restricted to predefined polymer spools, the modular stick-based concept enables greater flexibility in material selection and composite tuning. This is particularly relevant in biomedical research, where cytocompatible materials must be tested in limited batches, and the filament waste can be costly. Moreover, our system allows for consistent scaffold geometry and architecture, which is critical in studies assessing cell–material interactions. We believe this approach bridges a crucial gap between lab-scale material development and functional 3D printing for patient-specific applications.

### Limitations and Future Work

This study had several limitations that should be acknowledged. One notable setback was the use of literature-based material data rather than experimentally obtained data for pure PCL and its composites with carbon nanotubes. Ideally, the material properties should be determined through experimental characterization and then implemented into the simulation model. However, comprehensive material data for polymers intended for medical applications are scarce or highly limited in available simulation databases. Moreover, simulation parameters do not always fully reflect the real conditions of an injection molding process. Our equipment was laboratory-scale, which restricts certain parameters, such as injection pressure (maximum 130 MPa), and lacks mold heating or temperature control. Although the simulation was set with a mold temperature of 22 °C, in practice, the mold temperature approximates ambient conditions. Additionally, the laboratory scale of this study may limit the direct scalability of the results to industrial applications. Moreover, long-term mechanical behavior, degradation, and biocompatibility of the materials were not addressed and remain crucial for evaluating their applicability in biomedical contexts. Preliminary FDM printing trials were promising, but a more comprehensive analysis of printing quality and interlayer bonding is needed. Future work will focus on the experimental characterization of mechanical and thermal properties of the developed composites, improving the simulation accuracy, upgrading processing equipment to better match industrial conditions, and conducting in vitro studies to assess the biological performance of the printed scaffolds.

## 5. Conclusions

The performed simulations and experimental trials confirmed that the PCL nanocomposites containing 0.5 wt.% MWCNTs offer excellent injection molding characteristics and are promising candidates for use in modular 3D-printable biomedical components. While higher CNT contents can provide stronger functional enhancements, they introduce substantial processing challenges. Therefore, the 0.5 wt.% CNT serves as the most practical and effective target concentration for developing advanced customizable biomaterials for medical applications. While preliminary FDM printing trials were promising, a more in-depth analysis is still needed. Future work will focus on: (1) experimental characterization of the composites’ thermal properties, (2) performing spectroscopic and microscopic analyses (e.g., FTIR, SEM) to confirm material structure and nanofiller distribution, (3) enhancing simulation accuracy, and (4) conducting in vitro studies to assess the biological performance of the printed scaffolds.

## Figures and Tables

**Table 1:** Physicochemical properties of carbon nanotubes used to build the model, based on literature.

Fibers Parameter	Value	Literature
fiber percentage, [%]	0.5; 5; 10	
aspect ratio,	150	
elastic modulus, [MPa]	180,000	[36,37]
Poisson’s ratio, -	0.27	[38]
density, [Kg/m^3^]	1900	[39,40]

**Table 2:** Manufacturing parameters used for simulation.

Process Parameters	Value
ambient temperature, [°C]	22
melt temperature, [°C]	170
mold temperature, [°C]	22
injection pressure limit, [MPa]	129
filling time, [s]	1.2
packing time, [s]	2.5
cooling time, [s]	25

**Table 3:** Summary of simulation results for different CNT concentrations.

Process Parameters	PCL_0.5	PCL_5	PCL_10
Total filling time, [s]	1.2	1.2	3.6
Pressure at the packing, [MPa]	80	80	66
Flow front temperature, [°C]	170	170	170
Frozen areas	No	No	No
Simulated fiber orientation	Easy	Easy	Easy, Difficult, Warning

**Table 4:** Manufacturing parameters—experimental tests.

Process Parameters	Value
ambient temperature, [°C]	22
melt temperature, [°C]	175
mold temperature, [°C]	22
hydraulic pressure, [MPa]	127
cooling time, [s]	28

## Data Availability

The raw/processed data required to reproduce these findings cannot be shared at this time, as the data are also part of an ongoing study.

## Abbreviations

The following abbreviations are used in this manuscript:

MDPI	Multidisciplinary Digital Publishing Institute
DOAJ	Directory of open access journals
TLA	Three-letter acronym
LD	Linear dichroism
